# Bidirectional associations between sleep problems and behavioural difficulties and health‐related quality of life in adolescents: Evidence from the SCAMP longitudinal cohort study

**DOI:** 10.1002/jcv2.12098

**Published:** 2022-08-17

**Authors:** Chen Shen, Michael O. Mireku, Martina Di Simplicio, Iroise Dumontheil, Michael S. C. Thomas, Martin Röösli, Paul Elliott, Mireille B. Toledano

**Affiliations:** ^1^ Department of Epidemiology and Biostatistics MRC Centre for Environment and Health School of Public Health Imperial College London London UK; ^2^ National Institute for Health Research Health Protection Research Unit in Chemical and Radiation Threats and Hazards Imperial College London London UK; ^3^ School of Psychology University of Lincoln Lincoln UK; ^4^ Lincoln Sleep Research Centre University of Lincoln Lincoln UK; ^5^ Department of Brain Sciences Division of Psychiatry Imperial College London London UK; ^6^ Department of Psychological Sciences Birkbeck, University of London London UK; ^7^ Department of Epidemiology and Public Health Swiss Tropical and Public Health Institute Basel Switzerland; ^8^ Faculty of Science University of Basel Basel Switzerland; ^9^ National Institute for Health Research Biomedical Research Centre Imperial College London London UK; ^10^ Mohn Centre for Children's Health and Wellbeing School of Public Health Imperial College London London UK

**Keywords:** externalising difficulties, internalising difficulties, longitudinal studies, quality of life, sleep problems

## Abstract

**Background:**

Sleep problems show associations with negative outcomes in both physical and mental health in adolescents, but the associations may be reciprocal. We aimed to assess bidirectional associations between sleep problems and mental health symptoms including behavioural difficulties (internalising and externalising difficulties) and low health‐related quality of life (HRQoL).

**Methods:**

A total of 6616 adolescents (52.4% females) across Greater London completed baseline assessments when they were aged 11–12 years, and 3803 of them (57.2% females) completed follow‐up assessments at aged 13–15 years. Weekday and weekend sleep duration were derived from self‐reported bedtime, sleep onset latency and wake time. Sleep disturbance was assessed using a standardized sleep disturbance scale. Internalising and externalising difficulties were assessed using subscales of the Strength and Difficulties Questionnaire. HRQoL was assessed using the KIDSCREEN‐10 questionnaire. Cross‐lagged structural equation modelling was used with multiple imputation to examine bidirectional associations between sleep problems and mental health symptoms.

**Results:**

Females had greater internalising difficulties, worse HRQoL and more sleep disturbance than males. Persistent insufficient weekday and weekend sleep, and sleep disturbance (i.e., at both baseline and follow‐up) were associated with internalising and externalising difficulties and low HRQoL at follow‐up (ORs ranged from 1.53 to 3.63). Persistent externalising difficulties and low HRQoL were also associated with insufficient weekend sleep and sleep disturbance at follow‐up (ORs ranged from 1.68 to 4.25). Using continuous variables, we found bidirectional associations between weekday sleep duration and HRQoL, weekend sleep duration and externalising score, sleep quality and internalising score, and sleep quality and HRQoL. The association magnitudes were mostly similar in the two directions.

**Conclusions:**

Our study showed bidirectional associations between sleep problems and mental health symptoms during adolescence, indicating that early intervention and treatment on the first‐occurring symptom may prevent the development of subsequent problems.

## Graphical abstract

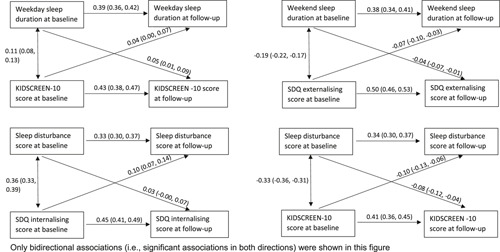

We assessed bidirectional associations between sleep problems and mental health symptoms including behavioural difficulties (internalising and externalising difficulties) and low health‐related quality of life (HRQoL). Bidirectional associations were found between weekday sleep and HRQoL, weekend sleep and externalising score, as well as sleep quality and internalising score and HRQoL.

**Key points**

- Sleep problems and mental health symptoms emerge during adolescence and are interrelated
- The associations between sleep problems and behavioural difficulties and low HRQoL have been found in previous studies with methodological and analytical limitations
- Our study found bidirectional associations between weekday sleep and HRQoL, weekend sleep and externalising score, as well as sleep quality and internalising score and HRQoL
- Our findings suggest that early intervention and treatment on the first‐occurring symptom may prevent the development of subsequent problems
- Future studies with longer follow‐up and more waves of longitudinal data could delineate the trajectories of the sleep and mental health problems during adolescence. Investigation of psychological factors related to sleep and mental health problems is also needed to reveal underlying mechanisms

**Abbreviations**
AIC: Akaike's information criterion
BIC: Bayesian information criterion
CFI: comparative fit index
HRQoL: health‐related quality of life
SCAMP: Study of Cognition, Adolescents and Mobile Phones
SDQ: Strengths and Difficulties Questionnaire
SOL: sleep onset latency
SRMR: standardized root‐mean‐square residual

## INTRODUCTION

Sleep is essential for adolescent development and maintenance of daily cognitive functioning such as emotional regulation, attention and impulse control, therefore restricting sleep can have many negative consequences (Dahl & Lewin, 2002; Gregory & Sadeh, 2012; Sadeghi‐Bahmani & Brand, 2022). The United States National Sleep Foundation recommends a minimum sleep duration of 9 h for school‐aged children (6–13 years‐old) and 8 h for teenagers (14–17 years‐old) (Hirshkowitz et al., 2015). In the UK, sleep deprivation is prevalent during adolescence, with one‐third of adolescents reporting less than 8 h of sleep per day (Conklin et al., 2018), and 6% having disturbed sleep (Dregan & Armstrong, 2010). The duration of sleep has also declined over the past 20 years (Keyes et al., 2015). Adolescence is a period when mental health problems begin to manifest (Kessler et al., 2005). Adolescent mental illness is a substantial public health burden in the UK, with one in seven 10–14 year‐olds and one in six 15–19 year‐olds suffering from mental health symptoms (Ferrari et al., 2016), which adversely influence social functioning, educational achievement and subsequent economic potential (Copeland et al., 2021; Copeland et al., 2015).

Sleep problems such as insufficient sleep duration, sleep disturbance, and sleep‐wake problems are associated with behavioural problems, self‐harm behaviours, depressive symptoms, anxiety, and worse health‐related quality of life (HRQoL) in adolescents (Bøe et al., 2012; Roeser et al., 2012; Shimizu et al., 2021; Shochat et al., 2014; Sivertsen et al., 2021; Sivertsen et al., 2015). Reversely, behavioural problems and emotional disturbance are associated with difficulties in sleep onset and maintenance (Ivanenko et al., 2005; Meijer et al., 2010), thereby indicating a potential bidirectional relationship between sleep and mental health. However, most studies are cross‐sectional and unable to disentangle such a cyclic pattern.

Some longitudinal studies concerning sleep and mental health in adolescents only investigated the role of sleep duration without further including sleep quality (Roane & Taylor, 2008; Roberts & Duong, 2013, 2014). Sleep quality reflects a subjective experience of sleep such as sleep depth, how well rested one feels after waking up, and general satisfaction with sleep (Pilcher et al., 1997). Poor sleep quality that is, sleep disturbance is particularly related to internalising problems and relatively independent of sleep duration (Meijer et al., 2010), perhaps because of the potential non‐linear association between sleep duration and psychological functioning (Kaneita et al., 2007). Moreover, the majority of longitudinal studies only considered a unidirectional relationship to investigate sub‐optimal sleep as a risk factor for development of mental health symptoms. Early childhood sleep disorders were associated with development of mental health problems in adolescence (Lam & Lam, 2021; Quach et al., 2009). To our knowledge, only a small number of adolescent cohort studies have explicitly investigated reciprocal associations between sleep and behavioural difficulties and HRQoL, with mixed results. One study (*n* = 555, aged 11–16 years at baseline) found that problems falling into sleep predicted internalising and externalising problems one year later, but not the reverse (Pieters et al., 2015). Two studies with long‐term follow‐up from childhood (aged 4–5 years) to adolescence (aged 12–14 years) showed that sleep problems were associated with later internalising difficulties (Quach et al., 2018; Wang et al., 2016), but only one found a marked reverse association (Wang et al., 2016). However, none of these three studies considered both sleep duration and quality. Third, most longitudinal studies did not distinguish between the role of sleep problem as a risk factor (i.e., present in a healthy individual months or years before the onset of mental health problems) or a concurrent manifestation of mental health problems (Fava & Mangelli, 2001), possibly resulting in misinterpretation of the findings. Thus, it is important to articulate the temporal relationship between sleep and mental health as well as the role of sleep problems on mental health symptoms through rigorous study design, measurements, and data analysis.

It has been well documented that females have a higher lifetime prevalence of internalising problems, but lower prevalence of externalising problems than males (Merikangas et al., 2010). Females are also more likely to report sleep disturbance during adolescence (aged 13–18 years) (Guo et al., 2014). Previous studies in children and adolescents (*N* < 400) did not find gender differences in the associations between sleep problems and behavioural difficulties or depressive and anxiety symptoms (Alfano et al., 2009; Wong et al., 2009), but these studies are relatively small‐scale, which might limit the power to detect potential effect modification by gender. Moreover, no previous studies have specifically examined whether gender modified bidirectional relationships between sleep and behavioural difficulties and HRQoL. Thus, addressing this uncertainty may shed some light on gender differences in the development of mental health problems starting from adolescence. By leveraging rich phenotyping data on sleep and mental health from the Study of Cognition, Adolescents and Mobile Phones (SCAMP), a recently established large adolescent cohort study in Greater London (Toledano et al., 2019), we could add to the literature by considering various sleep measures including weekday/weekend sleep duration and sleep quality when assessing the hypothesised bidirectional associations between sleep and mental health (internalising and externalising difficulties, and HRQoL). We also examined whether these associations varied by gender as another addition to previous studies.

## METHODS

### Participants

SCAMP is a prospective adolescent cohort study aimed at investigating cognitive and behavioural outcomes affected by use of mobile phones and other wireless technologies that emit radio frequency electromagnetic fields. Details of this study have been reported elsewhere (Toledano et al., 2019). We invited 206 schools across Greater London with Year 7 headcount *N* > 200 for state schools, and *N* > 50 for independent schools as the eligibility criteria. A total of 39 schools (26 state/13 independent) agreed to participate. Between November 2014 and July 2016 baseline data were collected from 6616 participants in Year 7 (aged 11–12 years). Of these participants, 3803 (retention rate 57.5%), from 31 (20 state/11 independent) schools, completed a follow‐up assessment between November 2016 and July 2018 when they were in Year 9/10 (aged 13–15 years).

Participants completed a computer‐based assessment via the Psytools software (Delosis Ltd, London), in examination mode. The assessment included a questionnaire on their digital technology behaviours (e.g., smartphone use, social network site engagement and video gaming), a battery of cognitive tests, and health, HRQoL and behaviour scales.

### Measures

#### Sleep

Participants were asked to report their usual bedtime, sleep onset latency (SOL), and wake time, for weekdays and weekends separately. Bedtime and wake times were provided as 30‐min interval categories (e.g. 08:30–09:00 p.m., 06:00–06:30 a.m.). For SOL, the following response categories were provided: “I fall asleep as soon as I get into bed”, “about 5 min”, “about 15 min”, “about 30 min”, “about 45 min”, “1–2 h”, “3 h or more”. We took lower category boundaries (e.g. 08:30 p.m. for bedtime, 06:00 a.m. for wake time, and 0 min for SOL) to derive sleep duration. Insufficient sleep duration was classified as sleep duration <9 h for participants under 14 years and <8 h for participants 14+ years (Hirshkowitz et al., 2015). The prevalence of insufficient weekday sleep was 49.7% at baseline and 52.4% at follow‐up. The prevalence of insufficient weekend sleep was 31.7% at baseline and 24.4% at follow‐up.

Sleep quality was assessed using a 4‐item self‐reported standardised sleep disturbance scale (Schmitt et al., 2000). Respondents scored their frequency of difficulty in falling asleep, restless sleep, waking up throughout the night and waking up too early in the morning using a four‐point Likert scale (summary score ranging from 0 to 12). The Cronbach's *a* for sleep disturbance scale was 0.71 at baseline and 0.73 at follow‐up. Sleep disturbance was classified as sleep disturbance score of 10 or higher (Frei et al., 2014). The prevalence of sleep disturbance was 7.8% at baseline and 6.8% at follow‐up.

#### Internalising and externalising difficulties

Behavioural difficulties were assessed by the self‐reported Strengths and Difficulties Questionnaire (SDQ), with higher scores indicating greater behavioural difficulties (Goodman et al., 2000; Ravens‐Sieberer et al., 2010). Specifically, internalising difficulties were assessed using the 10 items from the emotional and peer problems subscales (summary score ranging from 0 to 20). The Cronbach's *a* for SDQ emotional and peer problems subscales in our sample was 0.70 at baseline and 0.71 at follow‐up. A summary score of 10 or above (i.e., scores on or above borderline) was classified as the presence of internalising difficulties (Goodman et al., 1998). The prevalence of internalising difficulties was 8.5% at baseline and 10.0% at follow‐up. Externalising difficulties were assessed using the 10 items from the conduct and hyperactivity/inattention problems subscales (summary score ranging from 0 to 20). The Cronbach's *a* for SDQ conduct and hyperactivity/inattention problems subscales in our sample was 0.75 at both baseline and follow‐up. A summary score of 10 or above (i.e., scores on or above borderline) was classified as the presence of externalising difficulties (Goodman et al., 1998). The prevalence of externalising difficulties was 13.3% at baseline and 15.6% at follow‐up.

#### HRQoL

HRQoL was assessed with self‐reported 10‐item KIDSCREEN‐10; higher scores indicate better HRQoL (Ravens‐Sieberer et al., 2010). The Cronbach's *α* for KIDSCREEN‐10 in our sample was 0.74 at baseline and 0.78 at follow‐up. A summary score of 38 or below was classified as noticeable low HRQoL (Ravens‐Sieberer et al., 2013). The prevalence of low HRQoL was 6.9% at baseline and 12.4% at follow‐up.

The pattern of sleep problems and mental health symptoms was categorised into four groups according to whether symptoms were present (i.e., scores above the cut‐off) or absent (i.e., scores below the cut‐off) at baseline and follow‐up: no symptoms at baseline or follow‐up (reference group), symptoms at baseline only, symptoms at follow‐up only, and symptoms present at both timepoints (persistent group). We examined the associations between the pattern of sleep problems or mental health symptoms and the presence of mental health symptoms or sleep problems at follow‐up, respectively. We also examined the associations between sleep problems or mental health symptoms at baseline (regardless of follow‐up symptom status) and the presence of mental health symptoms or sleep problems at follow‐up, respectively.

#### Co‐variates

Demographic information including age, gender, ethnicity (combined into “White”, “Black”, “Asian”, “Mixed” and “Other” categories), parental education and parental occupation was captured in the SCAMP assessment. Parental education was categorised in a binary form as follows: at least one parent with higher education versus no parent with higher education. We used the Office for National Statistics classification of parental occupation, separating it into three levels. Each child was allocated the higher level of either parent.

### Statistical analysis

Chi‐square test was performed to compare sleep problems and mental health symptoms by gender. Paired *T*‐test was used to assess the changes in sleep problems and mental health symptoms from baseline to follow‐up, and ANOVA was used to assess the differences in such changes by gender.

Multivariable logistic regression was used to assess the associations between sleep and the development of mental health symptoms and vice versa. Potential confounders included age, gender, ethnicity, parental education, parental occupation, school type (state and independent), and mental health and sleep variables at baseline, selected based on directed acyclic graphs. For instance, we adjusted for socio‐demographic confounders and baseline summary score of SDQ emotional and peer problems subscales when assessing the association between sleep problems and the development of internalising difficulties at follow‐up. We also examined whether these associations were modified by gender from the significance of the interaction term.

We intended to assess the school clustering effect using mixed effect model with a random intercept for each school. Model fit was evaluated based on Akaike's information criterion (AIC), Bayesian information criterion (BIC), and deviance, with lower values indicating better fit. We compared AIC, BIC, and deviance between mixed effect models and models with fixed effect only. The inclusion of a random intercept did not improve model fit. Therefore, we did not repeat mixed effect model to maintain model parsimony.

To evaluate potential bidirectional relationships among sleep and mental health variables, we used cross‐lagged structural equation modeling to determine whether each of three sleep variables predicted each of three mental health measures or vice versa (nine models in total). Comparative fit index (CFI) and standardized root‐mean‐square residual (SRMR) were used to assess model fit with criteria of CFI ≥0.95 and SRMR ≤0.09 given our sample size (Hu & Bentler, 1999). All nine models had good fit. Wald tests were used to examine coefficient differences between pairs of bidirectional paths to indicate whether sleep driven paths or mental health driven paths were stronger. Standardized *ß* coefficients were computed to enable comparison of estimates between each direction. As a sensitivity analysis, we excluded participants (*n* = 206) with combined weekday and weekend sleep duration above the recommended level (i.e., >11 h for 6–13 year olds and >10 h for 14–17 year olds) (Hirshkowitz et al., 2015) to mitigate concerns that the potential adverse mental health effect of oversleep might affect the bidirectional relationships.

Given that loss of follow‐up mainly depended on factors that might cause school to drop out follow‐up assessment such as logistical issues with school timetables, we assumed that missingness was at random. To minimise potential biases due to loss of follow‐up and missing data, we used multiple imputation based on 5534 participants with complete baseline sleep and mental health data to predict missing ethnicity (1.6%), parental education (22.3%), parental occupation (5.3%), and follow‐up sleep and mental health variables (42.6%–48.5%) on all available cases (i.e., including those who were lost to follow‐up). Multiple imputation was based on a flexible additive regression model, incorporating data on weekday and weekend sleep duration, sleep quality, scores of four SDQ subscales and KIDSCREEN‐10 at baseline, gender, age, and school type. No additional auxiliary variables were included in the imputation model. We generated 20 imputed datasets and summarized the results from these datasets into single estimates with 95% confidence intervals (95% CIs) adjusted for missing data uncertainty. We also performed complete case analyses in the longitudinal sample (i.e., participants with completed data at baseline and follow‐up) without imputation as sensitivity analyses to examine whether differences in sample characteristics altered the results. All analyses were performed using STATA version IC/13.1 for Windows (Stata Corp, College station, Texas, USA).

## RESULTS

Table 1 shows the proportion of females was 52.4% at baseline and 57.2% at follow‐up. The median age (interquartile range) of our study sample was 12.06 (11.79, 12.33) years at baseline and 14.21 (13.92, 14.54) years at follow‐up. The sample was diverse in terms of socio‐demographic characteristics, although loss to follow‐up was slightly higher in disadvantaged groups.

**TABLE 1:** Socio‐demographics characteristics in the SCAMP cohort at baseline and follow‐up

	Baseline (*n* = 6616)	Follow‐up (*n* = 3803)
Age (years), median (IQR)	12.06 (11.79, 12.33)	14.21 (13.92, 14.54)
Sex, *n* (%)		
Male	3147 (47.6)	1627 (42.8)
Female	3469 (52.4)	2176 (57.2)
Ethnicity, *n* (%)		
White	2820 (42.7)	1709 (44.9)
Black	1016 (15.4)	565 (14.9)
Asian	1758 (26.6)	1024 (26.9)
Mixed	740 (11.2)	433 (11.4)
Others	62 (0.9)	36 (1.0)
Missing/not interpretable	220 (3.3)	36 (1.0)
Parental education, *n* (%)		
At least one	3677 (55.6)	2375 (62.5)
None	1200 (18.1)	800 (21.0)
Missing	1739 (26.3)	628 (16.5)
Parental occupation, *n* (%)		
Managerial/professional occupations	3426 (51.8)	2173 (57.1)
Intermediate occupations	1480 (22.4)	867 (22.8)
Routine and manual occupations	1040 (15.7)	609 (16.0)
Missing/not interpretable	670 (10.1)	154 (4.1)
Type of school, *n* (%)		
State	5141 (77.7)	2760 (72.6)
Independent	1475 (22.3)	1043 (27.4)

Abbreviation: IQR, Interquartile range.

Table 2 shows that the prevalence of weekday insufficient sleep was similar between males and females at baseline and follow‐up, but there was a higher rate of females who had internalising difficulties, low HRQoL and sleep disturbance emerging at follow‐up, or present at both timepoints (all *p* for Chi‐squared tests <0.001). Absolute gender differences in prevalence of these measures ranged from 1.3% to 12.0%. Correlation between sleep duration and quality was low (Spearman correlation coefficient ranged from −0.17 to −0.08). Table S1 shows a decline in sleep duration from baseline to follow‐up, particularly in females. Greater behavioural difficulties and lower HRQoL were also observed at follow‐up than baseline, particularly in females.

**TABLE 2:** The pattern of sleep problems and mental health symptoms at baseline and follow‐up by gender in the SCAMP cohort (*n* = 3803)

Symptoms	Timepoints	Males, *n* (%)	Females, *n* (%)	*p* value
Insufficient weekday sleep	None^a^	465 (31.3)	631 (30.5)	0.17
	Baseline only^b^	264 (17.8)	317 (15.3)	
	Follow‐up only^c^	306 (20.6)	460 (22.3)	
	Both^d^	451 (30.4)	659 (31.9)	
Insufficient weekend sleep	None	776 (52.2)	1312 (63.5)	<0.001
	Baseline only	309 (20.8)	287 (13.9)	
	Follow‐up only	185 (12.5)	263 (12.7)	
	Both	216 (14.5)	205 (9.9)	
Sleep disturbance	None	1318 (90.1)	1753 (85.5)	<0.001
	Baseline only	82 (5.6)	125 (6.1)	
	Follow‐up only	51 (3.5)	129 (6.3)	
	Both	12 (0.8)	44 (2.2)	
Internalising difficulties	None	914 (75.9)	874 (53.1)	<0.001
	Baseline only	99 (8.2)	164 (10.0)	
	Follow‐up only	119 (9.9)	361 (21.9)	
	Both	73 (6.1)	248 (15.1)	
Externalising difficulties	None	809 (67.1)	1170 (71.0)	0.01
	Baseline only	112 (9.3)	110 (6.7)	
	Follow‐up only	176 (14.6)	250 (15.2)	
	Both	108 (9.0)	117 (7.1)	
Low HRQoL	None	1118 (88.1)	1383 (80.1)	<0.001
	Baseline only	52 (4.1)	77 (4.5)	
	Follow‐up only	76 (6.0)	213 (12.3)	
	Both	23 (1.8)	54 (3.1)	

*Note*: *p* value for two‐sided Chi‐squared tests.

Abbreviation: HRQoL, health‐related quality of life.

^a^ no symptoms (e.g. insufficient weekday sleep) at baseline or follow‐up.

^b^ symptoms present at baseline only.

^c^ symptoms present at follow‐up only.

^d^ symptoms present at both baseline and follow‐up.

Table 3 shows that persistent insufficient sleep on weekdays was associated with internalising difficulties (OR = 1.73, 95% CI 1.27, 2.35), externalising difficulties (OR = 1.62, 95% CI 1.25, 2.11), and low HRQoL (OR = 1.78, 95% CI 1.32, 2.38) at follow‐up. Persistent insufficient weekend sleep and sleep disturbance were also associated with all mental health symptoms at follow‐up (ORs ranged from 1.53 to 3.63). Cross‐sectional associations (associations between sleep problems and mental health symptoms both at follow‐up) were generally stronger than longitudinal associations (associations between sleep problems at baseline and mental health symptoms at follow‐up).

**TABLE 3:** The associations between sleep problems and the presence of mental health symptoms at follow‐up using multiple imputation (*n* = 5534)

		Sleep problems			
	Mental health symptoms at follow‐up	Baseline^a^	Baseline only^b^	Follow‐up only^b^	Both^b^
		OR (95% CI)	OR (95% CI)	OR (95% CI)	OR (95% CI)
Insufficient weekday	Internalising difficulties	1.36 (1.07, 1.73)	1.24 (0.85, 1.81)	1.37 (0.91, 2.05)	1.73 (1.27, 2.35)
Sleep	Externalising difficulties	1.15 (0.94, 1.41)	1.12 (0.84, 1.51)	1.68 (1.27, 2.23)	1.62 (1.25, 2.11)
	Low HRQoL	1.31 (1.04, 1.65)	1.13 (0.80, 1.61)	1.46 (1.04, 2.03)	1.78 (1.32, 2.38)
Insufficient weekend	Internalising difficulties	1.06 (0.80, 1.39)	0.96 (0.68, 1.35)	1.64 (1.15, 2.34)	1.53 (1.01, 2.30)
Sleep	Externalising difficulties	1.36 (1.10, 1.69)	1.31 (1.02, 1.68)	1.90 (1.43, 2.52)	2.01 (1.52, 2.67)
	Low HRQoL	1.15 (0.89, 1.49)	0.99 (0.74, 1.33)	1.50 (1.07, 2.11)	1.69 (1.12, 2.54)
Sleep disturbance	Internalising difficulties	0.99 (0.68, 1.44)	0.88 (0.57, 1.36)	2.78 (1.81, 4.25)	2.03 (1.03, 3.99)
	Externalising difficulties	1.21 (0.90, 1.62)	1.16 (0.84, 1.62)	2.26 (1.50, 3.40)	1.85 (1.00, 3.43)
	Low HRQoL	1.45 (1.06, 1.99)	1.26 (0.86, 1.85)	3.78 (2.50, 5.72)	3.63 (1.98, 6.64)

*Note*: Adjusted for age, gender, ethnicity, parental education, parental occupation, school type, and scores of respective mental health scales at baseline.

Abbreviation: HRQoL, health‐related quality of life.

^a^ Baseline sleep problems (reference group: no baseline sleep problems).

^b^ The pattern of sleep problems at baseline and follow‐up (reference group: no sleep problems at baseline or follow‐up).

Table 4 shows that persistent internalising and externalising difficulties were associated with insufficient weekend sleep (OR = 1.93, 95% CI 1.18, 3.15 and OR = 1.68, 95% CI 1.20, 2.37, respectively) and sleep disturbance (OR = 3.62, 95% CI 2.13, 6.15 and OR = 2.33, 95% CI 1.44, 3.76, respectively) at follow‐up. Persistent low HRQoL was associated with insufficient weekend sleep (OR = 1.96, 95% CI 1.23, 3.14) and sleep disturbance (OR = 4.25, 95% CI 2.39, 7.55) at follow‐up.

**TABLE 4:** The associations between mental health symptoms and the presence of sleep problems at follow‐up using multiple imputation (*n* = 5534)

		Mental health symptoms			
	Sleep problems at follow‐up	Baseline^a^	Baseline only^b^	Follow‐up only^b^	Both^b^
		OR (95% CI)	OR (95% CI)	OR (95% CI)	OR (95% CI)
Internalising difficulties	Insufficient weekday sleep	0.87 (0.67, 1.15)	0.81 (0.59, 1.12)	1.36 (1.00, 1.84)	1.10 (0.71, 1.68)
	Insufficient weekend sleep	1.27 (0.94, 1.72)	1.09 (0.75, 1.60)	1.68 (1.22, 2.31)	1.93 (1.18, 3.15)
	Sleep disturbance	1.78 (1.25, 2.52)	1.41 (0.88, 2.25)	2.79 (1.81, 4.32)	3.62 (2.13, 6.15)
Externalising difficulties	Insufficient weekday sleep	0.96 (0.74, 1.24)	0.93 (0.69, 1.27)	1.72 (1.37, 2.15)	1.16 (0.79, 1.69)
	Insufficient weekend sleep	1.22 (0.95, 1.58)	1.10 (0.76, 1.57)	1.83 (1.39, 2.41)	1.68 (1.20, 2.37)
	Sleep disturbance	1.52 (1.10, 2.10)	1.35 (0.87, 2.08)	2.40 (1.57, 3.65)	2.33 (1.44, 3.76)
Low HRQoL	Insufficient weekday sleep	1.06 (0.81, 1.40)	0.95 (0.67, 1.33)	1.48 (1.11, 1.96)	1.49 (0.94, 2.36)
	Insufficient weekend sleep	1.47 (1.08, 2.01)	1.38 (0.93, 2.04)	1.67 (1.21, 2.32)	1.96 (1.23, 3.14)
	Sleep disturbance	1.67 (1.11, 2.52)	1.21 (0.65, 2.26)	3.68 (2.49, 5.42)	4.25 (2.39, 7.55)

*Note*: Adjusted for age, gender, ethnicity, parental education, parental occupation, school type, and respective continuous sleep variables at baseline.

Abbreviation: HRQoL, health‐related quality of life.

^a^ Baseline mental health symptoms (reference group: no baseline mental health symptoms).

^b^ The pattern of mental health symptoms at baseline and follow‐up (reference group: no mental health symptoms at baseline or follow‐up).

**FIGURE 1:**
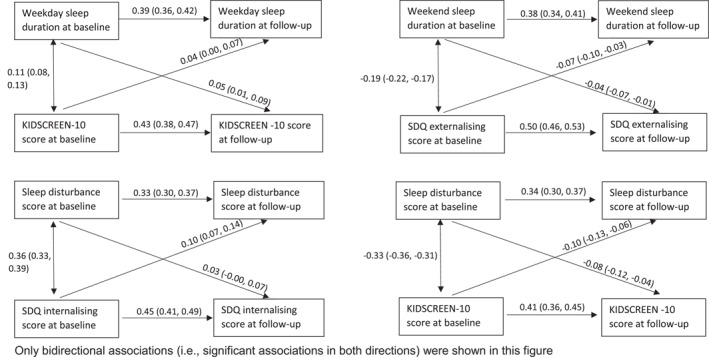
Bidirectional associations (β (95% CI)) between sleep problems and mental health symptoms using cross‐lagged structural equation modeling with multiple imputation (*n* = 5534)

Using continuous variables, bidirectional relationships were observed for weekday sleep duration and HRQoL, weekend sleep duration and externalising score, sleep quality and internalising score, and sleep quality and HRQoL (Figure 1). Association magnitudes were similar in each direction with the exception that the association between internalising score and subsequent sleep quality was stronger than the reverse. Sensitivity analyses by excluding participants with sleep duration above recommended levels yielded similar results (Figure S1).

Associations between different sleep characteristics and mental health symptoms were generally similar between males and females (all *p* values for interaction by gender >0.15). Complete case analyses in Tables S2 and S3 and Figure S2 showed similar results.

## DISCUSSION

Our study has shown persistent insufficient sleep and sleep disturbance were associated with internalising and externalising difficulties, and low HRQoL at follow‐up. Persistent internalising and externalising difficulties and low HRQoL were also associated with sleep disturbance and insufficient weekend sleep at follow‐up. Our results indicate bidirectional relationships between weekday sleep duration and HRQoL, weekend sleep duration and externalising score, as well as sleep quality and internalising score and HRQoL. No significant bidirectional relationships were found between other sleep and mental health variables. All associations were similar between males and females.

Our findings are in line with most of the existing longitudinal studies concerning sleep problems, which show these to be associated with later behavioural difficulties and HRQoL in adolescents (Pieters et al., 2015; Quach et al., 2018; Williamson et al., 2021). However, these studies only included one composite sleep measure. Our study adds to previous research by considering different sleep measures and showed that persistent insufficient sleep (specified by weekdays and weekends), and sleep disturbance were separately associated with internalising and externalising difficulties and low HRQoL at follow‐up after controlling for baseline mental health measures. Given the low correlation between sleep duration and quality observed in our study, multiple sleep measures may be required to have a comprehensive picture of an individual's sleep. To our knowledge, no previous studies have explicitly distinguished sleep problem as a risk factor or a concurrent manifestation for behavioural difficulties or low HRQoL. The stronger cross‐sectional associations than longitudinal associations suggest that sleep problem might be a concurrent manifestation of mental health problems developed in close temporal proximity. Given the development of mental health symptoms is rarely rapid in non‐clinical samples, sleep problem might be an important indicator of early stage mental health problems in adolescents.

Our study also found evidence that mental health symptoms including behavioural difficulties and low HRQoL could temporally precede the development of sleep problems, which is consistent with some studies showing bidirectional relationships between sleep quality and behavioural difficulties (Meijer et al., 2010; Vazsonyi et al., 2022). However, our findings are inconsistent with some other studies that showed sleep problems predicted behavioural difficulties, but not the other way around (Pieters et al., 2015; Quach et al., 2018). This is perhaps because these studies did not specify weekday and weekend sleep deprivation, where the drivers might be different. In our study, the associations between behavioural difficulties and subsequent insufficient sleep on weekends were generally stronger than that between behavioural difficulties and insufficient sleep on weekdays. Insufficient sleep on weekdays is likely due to various reasons other than behavioural difficulties, such as academic and extracurricular demands, limited parental monitoring, and fixed school start time. Those with insufficient sleep on weekdays need a catch‐up sleep on weekends/holidays to compensate for their sleep debt. However, insufficient sleep on weekends may be due to internalising (e.g. maladaptive emotional regulation) (Dahl & Lewin, 2002) and externalising difficulties (e.g. hyperactivity) (Hysing et al., 2016). Sleep disturbance is more prominently affected than sleep duration by mental health symptoms. Increased fear, vigilance and stress, which are the hallmarks of internalising difficulties, impact normal sleep onset and continuity (Dahl & Harvey, 2007; Lu et al., 2018). It is also possible that increased vigilance to external cues and internal thoughts may trigger worrying about anticipated negative events or rumination about negative past events, which are typically described as hindering falling asleep by individuals with emotional dysregulation.

Sleep disturbance, internalising difficulties, and low HRQoL were more prevalent in female adolescents, in accordance with previous research (Shochat et al., 2014). However, we did not observe any gender‐specific associations between sleep problems and behavioural difficulties or HRQoL. Given that females did not show higher prevalence of insufficient sleep than males in our study, sleep disturbance might play a more important role than sleep duration in explaining gender differences in mental health.

Our study has strengths and weaknesses. Our study is based on a large‐scale prospective cohort of adolescents with detailed data on sleep and mental health measures. This enables us to consider differential associations of sleep duration and quality with various mental health measures. The cohort is representative of school‐aged children across Greater London. The results remained similar in the longitudinal sample, indicating that sample differences in SES was unlikely to impact our conclusions. The longitudinal study design enables the investigation of bidirectional associations between sleep problems and behavioural difficulties and HRQoL to improve our understanding of the complex relationship between sleep and mental health. However, our participants were assessed at two timepoints only. Longer follow‐up with more waves of data collection is essential to depict the developmental trajectories of sleep and mental health over the adolescence period. As in many epidemiological studies, mental health symptoms such as internalising and externalising difficulties were derived from self‐reported instruments rather than a clinical interview to define and classify mental disorders. We only assessed sleep duration and night‐time symptoms of sleep disturbance, without considering other modules on DSM‐V insomnia disorder or other sleep disorders (e.g. daytime fatigue). Thus, we were unable to examine the role of other comorbid sleep problems. The use of self‐reported questionnaires to collect information on sleep in our study is subject to recall bias and social desirability bias. In the future, wearable devices, such as actigraphy, could be used to measure sleep objectively and comprehensively. Finally, we could not fully control for residual confounding (e.g. neurological disorders and/or learning disabilities) due to the nature of observational study design, although we mitigated this limitation by adjusting for confounders rigorously on a scientific basis.

Our study has multiple implications. First, our findings showed a decrease in sleep duration and increase in mental health problems during the transition period from late childhood to adolescence, in line with epidemiological data indicating that mental health problems onset increases through adolescence (Kessler et al., 2005). This highlights the needs for more research to explore the drivers (e.g. social media use) (Foerster et al., 2019) and early intervention to tackle these common adolescent problems. Second, sleep problem might be a possible target for early identification and treatment of mental illness. Third, the bidirectional associations between sleep problems and mental health symptoms suggest that interventions focused on sleep might also benefit mental health, whilst interventions focused on mental health might also improve sleep. More in‐depth research in the psychological mechanisms (e.g. stress, rumination) is warranted to have a better understanding of these adolescent problems.

## CONCLUSIONS

This is a large longitudinal study following participants from late childhood to adolescence, a period during which mental health problems begin to emerge. Our analysis showed a bidirectional association pattern between sleep problems and mental health symptoms, indicating that early intervention and treatment on the first‐occurring symptom may prevent the development of subsequent problems. Future large‐scale studies with longer follow‐up and more waves of longitudinal data could delineate the developmental trajectories of sleep and mental health problems during adolescence. Further investigation of psychological factors in relation to sleep and mental health problems is needed to reveal underlying mechanisms.

## AUTHOR CONTRIBUTIONS

**Chen Shen**: Conceptualization, Data curation, Formal analysis, Investigation, Methodology, Project administration, Visualization, Writing – original draft, Writing – review & editing. **Michael Mireku**: Formal analysis, Investigation, Methodology, Validation, Writing – review & editing; **Martina Di Simplicio**: Formal analysis, Methodology, Supervision, Validation, Writing – review & editing: **Iroise Dumontheil**: Funding acquisition, Project administration, Supervision, Writing – review & editing; **Michael Thomas**: Funding acquisition, Project administration, Supervision, Writing – review & editing; **Martin Röösli**: Funding acquisition, Project administration, Supervision, Writing – review & editing; **Paul Elliott**: Conceptualization, Funding acquisition, Methodology, Project administration, Supervision, Writing – review & editing; **Mireille Toledano**: Conceptualization, Funding acquisition, Investigation, Methodology, Project administration, Supervision, Validation, Writing – review & editing.

## CONFLICTS OF INTEREST

The authors have declared that they have no competing or potential conflicts of interest.

## ETHICAL CONSIDERATIONS

The North West Haydock Research Ethics Committee approved the SCAMP study protocol and subsequent amendments (ref 14/NW/0347). School head teachers consented to participation in SCAMP. Parents and adolescents were provided in advance with written information about the study and were given the opportunity to opt out of the research at any time. The study was conducted in accordance with the Declaration of Helsinki.

## Supporting information

Supplementary Material

## Data Availability

The data that support the findings of this study are available on request from the corresponding author. The data are not publicly available due to SCAMP privacy policy and ethical restrictions. More information on data application process can be found on the study website (https://www.scampstudy.org/research‐opportunities/).
