# Supplementary material for: Bidirectional associations between sleep problems and behavioural difficulties and health‐related quality of life in adolescents: Evidence from the SCAMP longitudinal cohort study
Source: JCPP Adv. 2022 Aug 17;2(3):e12098. doi: 10.1002/jcv2.12098 (PMC10021029; doi:10.1002/jcv2.12098)
Supplement: Supplementary file 1 — Supplementary Material [file JCV2-2-e12098-s001.docx]

Table S1 Gender-specific changes in sleep and mental health between baseline and follow-up (as continuous variables) in the SCAMP cohort (n=3803)

|  |  | Male | Female | P value^b^ |
| --- | --- | --- | --- | --- |
| Weekday sleep duration (h), mean (SD) | Baseline | 8.77 (1.37) | 8.88 (1.18) |  |
|  | Follow-up | 8.08 (1.35) | 8.00 (1.32) |  |
|  | P value^a^ | <0.001 | <0.001 | <0.001 |
| Weekend sleep duration (h), mean (SD) | Baseline | 9.28 (1.84) | 9.75 (1.72) |  |
|  | Follow-up | 9.01 (1.89) | 9.31 (1.75) |  |
|  | P value | <0.001 | <0.001 | 0.008 |
| Sleep disturbance score, mean (SD) | Baseline | 4.74 (2.95) | 4.94 (2.97) |  |
|  | Follow-up | 3.98 (2.83) | 5.01 (3.02) |  |
|  | P value | <0.001 | 0.41 | <0.001 |
| SDQ internalising score, mean (SD) | Baseline | 2.34 (2.06) | 3.08 (2.26) |  |
|  | Follow-up | 2.41 (2.04) | 3.74 (2.44) |  |
|  | P value | 0.22 | <0.001 | <0.001 |
| SDQ externalising score, mean (SD) | Baseline | 5.53 (3.43) | 4.94 (3.33) |  |
|  | Follow-up | 6.07 (3.38) | 5.85 (3.52) |  |
|  | P value | <0.001 | <0.001 | 0.01 |
| KIDSCREEN-10 score, mean (SD) | Baseline | 49.37 (8.00) | 48.96 (8.51) |  |
|  | Follow-up | 47.43 (7.86) | 44.93 (7.69) |  |
|  | P value | <0.001 | <0.001 | <0.001 |

SDQ: Strengths and Difficulties Questionnaire; SD: standard deviation

a: P value for changes between baseline and follow-up

b: P value for gender differences in changes between baseline and follow-up

Table S2 The associations between sleep problems and the presence of mental health symptoms at follow-up using complete case analysis (n=2841)

|  |  | Sleep problems | | | |
| --- | --- | --- | --- | --- | --- |
|  | Development of mental health symptoms at follow-up | Baseline^a^ | Baseline only^b^ | Follow-up only^b^ | Both^b^ |
|  |  | OR (95% CI) | OR (95% CI) | OR (95% CI) | OR (95% CI) |
| Insufficient weekday | Internalising difficulties | 1.51 (1.16, 1.98) | 1.46 (0.97, 2.22) | 1.42 (0.95, 2.13) | 1.92 (1.36, 2.71) |
| sleep | Externalising difficulties | 1.21 (0.96, 1.51) | 1.31 (0.91, 1.88) | 1.84 (1.33, 2.55) | 1.72 (1.28, 2.31) |
|  | Low HRQoL | 1.35 (1.06, 1.71) | 1.20 (0.82, 1.75) | 1.42 (1.00, 2.01) | 1.77 (1.31, 2.40) |
|  |  |  |  |  |  |
| Insufficient weekend | Internalising difficulties | 1.08 (0.81, 1.43) | 1.05 (0.72, 1.53) | 1.85 (1.30, 2.64) | 1.53 (1.03, 2.27) |
| sleep | Externalising difficulties | 1.42 (1.12, 1.80) | 1.48 (1.09, 2.00) | 2.01 (1.47, 2.75) | 1.94 (1.40, 2.69) |
|  | Low HRQoL | 1.20 (0.93, 1.54) | 1.01 (0.72, 1.41) | 1.46 (1.05, 2.04) | 1.77 (1.27, 2.49) |
|  |  |  |  |  |  |
| Sleep disturbance | Internalising difficulties | 0.94 (0.62, 1.44) | 0.90 (0.54, 1.48) | 3.18 (2.08, 4.86) | 1.78 (0.85, 3.75) |
|  | Externalising difficulties | 1.16 (0.81, 1.67) | 1.18 (0.78, 1.79) | 2.44 (1.64, 3.63) | 1.55 (0.77, 3.12) |
|  | Low HRQoL | 1.47 (1.03, 2.11) | 1.36 (0.89, 2.09) | 3.95 (2.72, 5.75) | 3.26 (1.73, 6.15) |

HRQoL: health-related quality of life

Adjusted for age, gender, ethnicity, parental education, parental occupation, school type, and scores of respective mental health scales at baseline

a: Baseline sleep problems (reference group: no baseline sleep problems)

b: The pattern of sleep problems at baseline and follow-up (reference group: no sleep problems at baseline or follow-up)

Table S3 The associations between mental health symptoms and the presence of sleep problems at follow-up using complete case analysis (n=2841)

|  |  | Mental health symptoms | | | |
| --- | --- | --- | --- | --- | --- |
|  | Development of sleep problems at follow-up | Baseline^a^ | Baseline only^b^ | Follow-up only^b^ | Both^b^ |
|  |  | OR (95% CI) | OR (95% CI) | OR (95% CI) | OR (95% CI) |
| Internalising difficulties | Insufficient weekday sleep | 0.74 (0.56, 0.99) | 0.67 (0.46, 0.97) | 1.42 (1.04, 1.94) | 0.87 (0.52, 1.47) |
|  | Insufficient weekend sleep | 1.19 (0.88, 1.61) | 1.02 (0.68, 1.52) | 1.79 (1.30, 2.46) | 1.88 (1.12, 3.16) |
|  | Sleep disturbance | 1.99 (1.35, 2.93) | 1.83 (1.09, 3.09) | 3.20 (2.09, 4.91) | 4.02 (2.21, 7.30) |
|  |  |  |  |  |  |
| Externalising difficulties | Insufficient weekday sleep | 0.80 (0.62, 1.02) | 0.98 (0.69, 1.38) | 1.96 (1.50, 2.57) | 0.83 (0.56, 1.22) |
|  | Insufficient weekend sleep | 1.13 (0.87, 1.46) | 1.14 (0.79, 1.64) | 1.88 (1.44, 2.46) | 1.47 (0.98, 2.19) |
|  | Sleep disturbance | 1.40 (0.96, 2.05) | 1.68 (0.98, 2.87) | 2.72 (1.83, 4.04) | 2.07 (1.21, 3.54) |
|  |  |  |  |  |  |
| Low HRQoL | Insufficient weekday sleep | 1.04 (0.77, 1.42) | 0.94 (0.63, 1.39) | 1.48 (1.13, 1.94) | 1.31 (0.78, 2.19) |
|  | Insufficient weekend sleep | 1.44 (1.06, 1.98) | 1.30 (0.86, 1.96) | 1.73 (1.31, 2.28) | 1.61 (0.96, 2.71) |
|  | Sleep disturbance | 1.55 (1.02, 2.35) | 1.02 (0.52, 2.00) | 3.78 (2.62, 5.45) | 3.79 (2.13, 6.73) |

HRQoL: health-related quality of life

Adjusted for age, gender, ethnicity, parental education, parental occupation, school type, and respective continuous sleep variables at baseline

a: Baseline mental health symptoms (reference group: no baseline mental health symptoms)

b: The pattern of mental health symptoms at baseline and follow-up (reference group: no mental health symptoms at baseline or follow-up)

Figure S1 Bidirectional associations (β (95% CI)) between sleep problems and mental health symptoms using cross-lagged structural equation modeling with multiple imputation (n=5328)

0.39 (0.35, 0.42)

0.41 (0.37, 0.45)

Weekend sleep duration at follow-up

Weekend sleep duration at baseline

Weekday sleep duration at follow-up

Weekday sleep duration at baseline

-0.06 (-0.10, -0.03)

0.04 (0.00, 0.08)

0.12 (0.09, 0.14)

-0.20 (-0.23, -0.18)

0.04 (-0.00, 0.08)

-0.04 (-0.08, -0.00)

0.50 (0.46, 0.54)

0.43 (0.39, 0.46)

SDQ externalising score at baseline

KIDSCREEN -10 score at follow-up

SDQ externalising score at follow-up

KIDSCREEN-10 score at baseline

0.35 (0.31, 0.38)

0.34 (0.30, 0.37)

Sleep disturbance score at follow-up

Sleep disturbance score at baseline

Sleep disturbance score at follow-up

Sleep disturbance score at baseline

-0.09 (-0.13, -0.06)

0.11 (0.08, 0.15)

-0.33 (-0.36, -0.31)

0.36 (0.33, 0.39)

-0.08 (-0.12, -0.05)

0.04 (0.00, 0.07)

0.40 (0.37, 0.44)

0.46 (0.43, 0.50)

KIDSCREEN -10 score at follow-up

SDQ internalising score at follow-up

KIDSCREEN-10 score at baseline

SDQ internalising score at baseline

Participants with sleep duration above the recommended level was excluded (n=206)

Only bidirectional associations (i.e., significant associations in both directions) were shown in this figure

Figure S2 Bidirectional associations (β (95% CI)) between sleep problems and mental health symptoms using cross-lagged structural equation modeling with complete case analysis (n=2841)

0.35 (0.32, 0.38)

0.36 (0.33, 0.39)

Weekend sleep duration at follow-up

Weekend sleep duration at baseline

Weekday sleep duration at follow-up

Weekday sleep duration at baseline

-0.06 (-0.09, -0.02)

0.04 (0.01, 0.07)

-0.15 (-0.19, -0.12)

0.13 (0.09, 0.16)

-0.04 (-0.07, -0.01)

0.05 (0.02, 0.08)

0.48 (0.45, 0.51)

0.41 (0.38, 0.44)

SDQ externalising score at baseline

KIDSCREEN -10 score at follow-up

SDQ externalising score at follow-up

KIDSCREEN-10 score at baseline

0.33 (0.30, 0.37)

0.33 (0.29, 0.36)

Sleep disturbance score at follow-up

Sleep disturbance score at baseline

Sleep disturbance score at follow-up

Sleep disturbance score at baseline

-0.09 (-0.12, -0.05)

0.10 (0.06, 0.13)

-0.36 (-0.39, -0.33)

0.38 (0.35, 0.41)

-0.08 (-0.12, -0.05)

0.04 (0.00, 0.07)

0.39 (0.35, 0.42)

0.44 (0.41, 0.48)

KIDSCREEN -10 score at follow-up

SDQ internalising score at follow-up

KIDSCREEN-10 score at baseline

SDQ internalising score at baseline

Only bidirectional associations (i.e., significant associations in both directions) were shown in this figure
